# How openness to experience drives R&D staff’s innovative behavior: a nonlinear mediation and moderation perspective on flow experience and emotional intelligence

**DOI:** 10.3389/fpsyg.2025.1546608

**Published:** 2025-08-01

**Authors:** Yanqiong Liu, Zhaogang Sun

**Affiliations:** School of Business, Zhengzhou University of Aeronautics, Zhengzhou, China

**Keywords:** openness, innovation behavior, flow experience, emotional intelligence, nonlinear mediation

## Abstract

**Introduction:**

Openness to experience is widely recognized as a driver of innovation, yet how flow experience mediates this relationship and whether emotional intelligence (EI) moderates it remain underexplored.

**Methods:**

This study investigates the nonlinear mediating role of flow between openness and innovation behavior, and EI’s moderating effect, using quantitative data from 475 R&D professionals (male, 75.16%; female, 24.84%).

**Results:**

Results reveal a curvilinear mediation: moderate flow enhances innovation, but excessive flow diminishes it. EI buffers this relationship, enabling sustained innovation under high flow intensity.

**Discussion:**

The findings highlight the need to manage flow dynamics and underscore EI as a critical factor for fostering innovation in R&D contexts, offering practical insights for organizational creativity management.

## 1 Introduction

In today’s rapidly evolving work environment, fostering employee innovative behavior has emerged as a critical organizational capability for sustaining competitive advantage (De Jong and Den Hartog, 2010). Following Scott and Bruce's (1994) seminal definition, innovative behavior encompasses “the generation, introduction, and application of novel ideas to create value for organizations,” a concept fundamentally distinct from general creativity through its emphasis on implementation (Amabile, 2018). While Schumpeter’s (1934) foundational theory positioned innovation as the engine of economic development, contemporary organizational research increasingly recognizes that sustained innovation requires systematic cultivation at the individual level (Anderson and West, 1998). As markets grow increasingly complex and dynamic, understanding the antecedents of individual innovative behavior becomes imperative for organizations seeking to institutionalize innovation processes.

Among individual traits, openness to experience has emerged as a key driver of individual innovation. Openness reflects a person’s willingness to embrace new experiences, explore diverse ideas, and adapt to change—traits that encourage involvement in innovative activities (Lim, 2019). Research shows that individuals high in openness tend to be more creative and effective at problem-solving, allowing them to tackle complex challenges through novel solutions (Dai et al., 2024). However, while openness is essential, it cannot fully explain innovative behavior on its own. Other psychological factors, such as flow experiences and emotional intelligence, also play critical roles in fostering innovation. These traits work synergistically to enhance creativity, emotional regulation, and problem-solving, especially in uncertain or dynamic environments (Jafri et al., 2016; Thajil and Al-Abrrow, 2024).

Flow is a deep state of concentration where individuals become fully absorbed in their tasks, optimizing creativity and productivity (Wang and Shaheryar, 2020). When individuals experience flow, they perform at their best and engage deeply in creative processes. Studies indicate that flow not only enhances innovation but also encourages the search for novel solutions. However, the relationship between flow and innovation is not always linear. According to activation theory, moderate levels of stimulation and stress promote optimal performance, while excessive stimulation can impair performance. This implies that the relationship between flow and innovation is U-shaped: moderate flow fosters innovation, but excessive flow may cause individuals to miss critical environmental cues that could spark innovation (McHugh, 2016).

Despite its importance, research has largely treated flow as a linear facilitator of innovation, overlooking its potential nonlinear effects. Additionally, the role of flow as a mediator between openness and innovation remains underexplored. Emotional intelligence (EI), which refers to the multidimensional ability to “perceive, regulate, and manage emotions across four dimensions—self-emotional appraisal, others’ emotional appraisal, emotion regulation, and use of emotions” (Wong and Law, 2002), building on Mayer et al. (2001) foundational model and Goleman’s (1996) framework on emotional regulation in organizational contexts, may play a significant role in regulating the intensity of flow and enhancing innovation. By moderating the intensity of flow, emotional intelligence can extend the positive effects of openness and reduce any negative impact that flow might have on innovation (Jafri et al., 2016).

Emotional intelligence may also influence the nonlinear relationship between flow and innovation by adjusting the point at which excessive flow becomes detrimental. For instance, individuals with higher emotional intelligence are better able to manage their flow states, ensuring that innovation continues even in high-stress environments (Dai et al., 2024). This suggests that emotional intelligence is critical in sustaining innovation over time. While emotional intelligence has been shown to moderate creativity in other contexts, its role in the openness-flow-innovation relationship remains understudied (Thajil and Al-Abrrow, 2024; Wang and Shaheryar, 2020).

**Figure 1:**
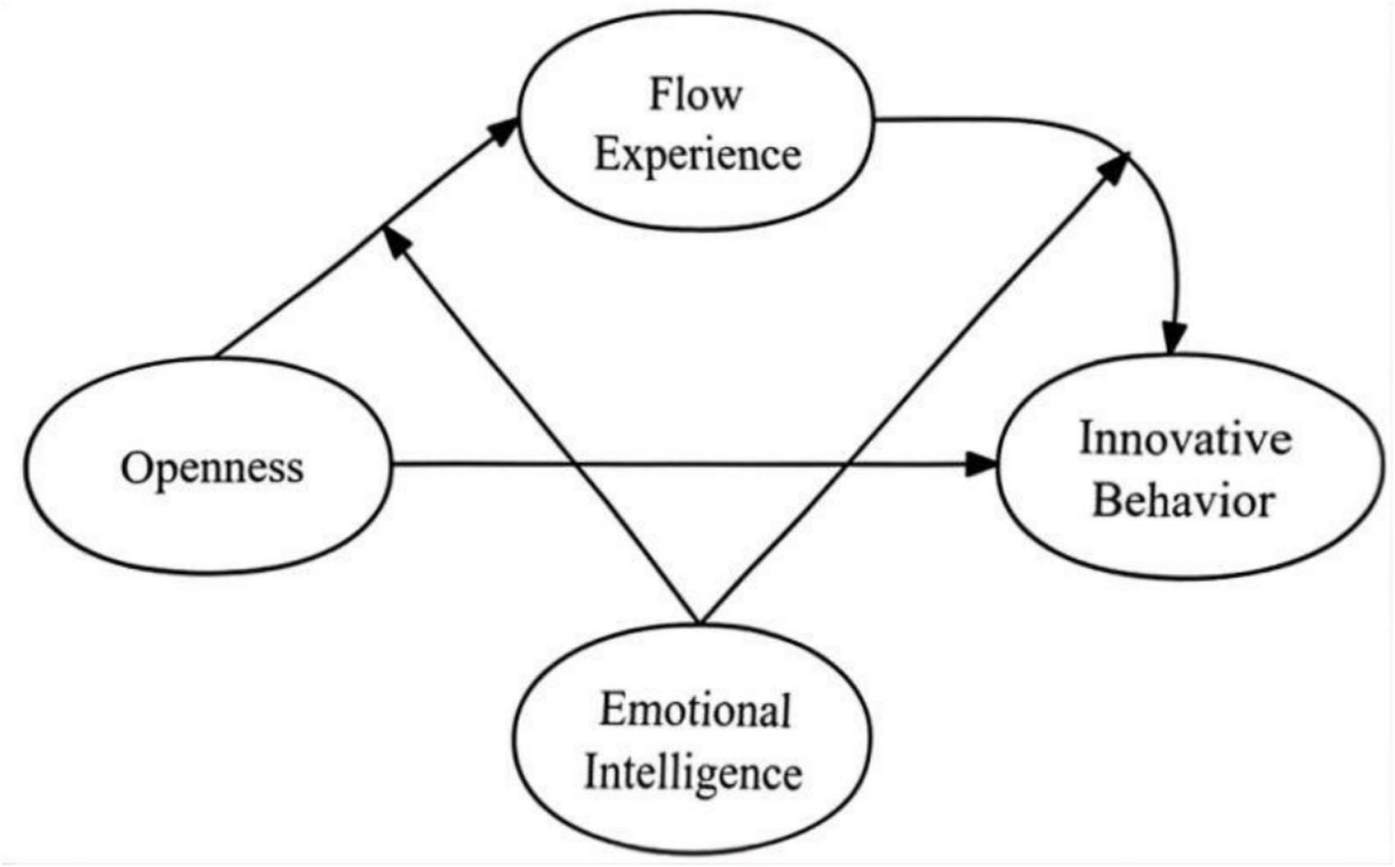
Theoretical model.

The goal of this study is to fill these research gaps by examining the relationship between openness and innovation through the nonlinear mediation of flow, as well as exploring the moderating effects of emotional intelligence on the curvilinear relationship between flow and innovation. The study makes several key contributions to the literature. First, it expands our understanding of how personality traits, particularly openness, drive innovation by considering the nonlinear mediation of flow. Second, it is the first to investigate the moderating role of emotional intelligence in the flow-innovation relationship, providing new insights into how organizations can further develop their employees’ innovative potential. Most importantly, this integrative model offers a fresh perspective on how innovative behavior occurs, providing practical insights for organizations aiming to foster innovation and enhance emotional intelligence among R&D personnel (Figure 1).

## 2 Theory and hypotheses

### 2.1 Theoretical perspective

#### 2.1.1 Flow theory

Flow theory, as introduced by Czikszentmihalyi (1990), described a deeply focused state where individuals become fully absorbed in their activities, achieving peak performance and intrinsic rewards. This concept is particularly relevant to creative tasks like innovation, which require intense concentration. In research and development (R&D) roles and other innovation-driven fields, flow states play a crucial role in problem-solving and generating new ideas (Czikszentmihalyi, 1990). However, recent studies suggest that the benefits of flow are not linear. Excessive flow can lead to cognitive fixation, limiting the ability to explore alternative solutions (Cseh et al., 2015). Research also indicates that moderate levels of flow optimize creativity, whereas excessive immersion may hinder adaptability and innovation (Goleman, 1996). These findings support the idea that the relationship between flow and innovation is nonlinear, with optimal innovation being achieved through moderate flow experiences rather than overwhelming immersion.

#### 2.1.2 Activation theory

Activation Theory (Gardner and Cummings, 1988), which aligns with Flow Theory, posits that individual performance peaks at an optimal level of arousal or activation. The Yerkes-Dodson Law asserts that moderate levels of arousal enhance performance, whereas very high or low levels can impair it. This theory helps explain the nonlinear relationship between flow and innovation, where moderate levels of activation during flow contribute to creativity and problem-solving, while excessive arousal impairs cognitive flexibility. Recent studies support this perspective, demonstrating that R&D professionals perform best when they are in a balanced state of flow with optimal levels of arousal (Roberts et al., 2014; Schutte and Malouff, 2020). These findings underscore that Activation Theory provides important insights into why flow may have curvilinear effects on innovation.

Activation Theory, introduced by Gardner and Cummings (1988), aligns closely with Flow Theory and suggests that individuals achieve peak performance at an optimal level of arousal or activation. Similarly, the Yerkes-Dodson Law proposes that moderate levels of arousal enhance performance, whereas very high or very low levels can impair it. This framework helps explain the nonlinear relationship between flow and innovation. Moderate levels of activation during flow enhance creativity and problem-solving abilities, while excessive arousal can hinder cognitive flexibility. Recent research supports this view, showing that R&D professionals perform at their best when they experience a balanced state of flow with optimal levels of arousal (Schutte and Malouff, 2020). These findings underscore the value of Activation Theory in providing insights into why flow can have curvilinear effects on innovation.

### 2.2 The nonlinear mediation role of flow experience

Flow experience plays a pivotal role in mediating the relationship between openness and innovative behavior. Drawing from Self-Determination Theory (SDT) and Activation Theory, it is hypothesized that the impact of flow on innovation follows a nonlinear pattern. Individuals who exhibit high levels of openness are naturally inclined to engage with new and challenging tasks, which makes it easier for them to enter flow states (Ryan and Deci, 2024). However, excessive flow can lead to cognitive narrowing, where individuals become so absorbed that they fail to explore alternative solutions, ultimately reducing their creative output (McHugh, 2016). This nonlinear dynamic is supported by empirical studies, which have demonstrated an inverted U-shaped relationship between flow intensity and creative performance (Goleman, 1996). Based on these findings, we propose the following hypothesis:

*H1*: Flow experience mediates the relationship between openness and innovative behavior in a nonlinear way, with moderate levels of flow enhancing innovation while excessive levels hinder it.

### 2.3 Emotional intelligence as a moderator

Emotional Intelligence (EI), which refers to the ability to perceive, regulate, and manage one’s emotions, plays a crucial role in managing both the cognitive and emotional challenges associated with innovative tasks. In the context of flow and Openness, EI serves as a moderator, enhancing individuals’ ability to stay focused and effectively navigate complex tasks. Those with high EI are better equipped to regulate their emotions and maintain flow states, which are vital for sustaining high performance levels (Goleman, 1996). Recent studies indicate that individuals with higher emotional intelligence are more adept at managing the emotional demands of complex tasks, enabling them to experience flow and maintain optimal performance even under high pressure (Malik, 2022; Rakei et al., 2022). These findings emphasize the moderating role of EI, particularly in individuals with high Openness, helping them maintain a balanced flow state and prevent the negative effects of excessive immersion. Based on these insights, we propose two hypotheses:

*H2a*: Emotional intelligence moderates the relationship between openness to flow experience, such that individuals with higher emotional intelligence are more likely to experience flow in response to openness.

*H2b*: Emotional intelligence moderates the nonlinear relationship between flow experience and innovation behavior, such that individuals with higher emotional intelligence sustain innovation performance at higher levels of flow, maintaining high performance even as flow intensity increases.

## 3 Methods

### 3.1 Sample and procedure

Data for this study were collected from R&D professionals working in innovation-driven industries such as technology, pharmaceuticals, and engineering. These sectors were chosen for their strong focus on creativity and problem-solving, which aligns with the study’s emphasis on openness, flow experience, innovative behavior, and emotional intelligence. To ensure a diverse sample, participants were randomly selected in collaboration with the HR departments of various organizations, with each participant having a minimum of 3 years of R&D experience. A total of 475 completed questionnaires were returned and validated for analysis, ensuring a sample size far exceeding the 20–300 participant threshold recommended for structural equation modeling (SEM) and Bootstrap methods to maintain statistical power (Hair et al., 2013). This large sample size effectively mitigates the risk of Type II errors and enhances result credibility. Additionally, given the challenges of accessing high-quality samples in R&D due to industry-specific constraints (e.g., corporate confidentiality, dispersed professional roles), the sample strategically covers multiple innovation-intensive sectors and leverages HR department collaboration to ensure data diversity, ensuring a broad representation across different sectors.

The demographic breakdown (see Table 1) highlights that 75.16% of participants were male, and 24.84% were female. In terms of age distribution, 44.21% of respondents were between 40 and 49 years, followed by 35.79% in the 20–29 years category. Regarding educational background, 48.21% held a master’s degree, 45.26% held a bachelor’s degree, and 6.53% had a doctorate. In terms of work experience, 35.79% of participants had 3–5 years of experience, while 21.05% had 16–20 years of experience. Most respondents held junior titles (35.79%), with a significant portion also holding associate senior titles (23.16%). Additionally, the majority of participants (50.53%) were employed in large organizations with over 1,000 employees, and 68.42% reported working between 40 and 59 h per week.

**Table 1:** Demographic characteristics of the sample.

Variable	Category	Count	Percentage (%)
Sex	Male	357	75.16
	Female	118	24.84
Age	20–29	170	35.79
	30–39	95	20
	40–49	210	44.21
Education	Bachelor’s	215	45.26
	Master’s	229	48.21
	Doctorate	31	6.53
Work Experience	3–5 years	170	35.79
	6–10 years	45	9.47
	11–15 years	75	15.79
	16–20 years	100	21.05
	21 years and above	85	17.89
Title	Junior title	170	35.79
	Intermediate title	80	16.84
	Associate senior title	110	23.16
	Senior title	80	16.84
	Others (please specify)	35	7.37
Unit Size	Below 50 people	40	8.42
	50–199 people	85	17.89
	200–499 people	40	8.42
	500–999 people	70	14.74
	1,000 people and above	240	50.53
Work Hours	Below 20 h	25	5.26
	20–39 h	65	13.68
	40–59 h	325	68.42
	60 h and above	60	12.63

### 3.2 Measures

#### 3.2.1 Openness

The Openness variable was measured using two items from the Openness subscale of the Big Five Inventory. These items assess an individual’s openness to new experiences, creativity, and intellectual curiosity. Sample items include: “I often try out new things and have original ideas” and “I follow routines and avoid innovation” (reverse-scored). The internal consistency of these two items was acceptable, with a Cronbach’s *α* of 0.767. Previous studies have found that the Openness factor is moderately correlated with Extraversion, as both traits are related to cognitive engagement and exploratory behavior (McCrae and Costa Jr, 1997). To further validate the construct, this study used Extraversion as a criterion variable to examine the criterion-related validity of the Openness scale. The Pearson correlation coefficient between Openness and Extraversion was *r* = 0.51, indicating good criterion validity. This suggests that the Openness subscale is appropriate for measuring individual differences in openness in the current sample.

#### 3.2.2 Flow experience

The Flow Experience variable was assessed using an adapted version of the flow scale by Wu et al. (2020), which was modified to suit the R&D context. One sample item is “I often become deeply immersed in R&D work,” reflecting the core dimension of task absorption. The scale exhibited excellent internal consistency, with a Cronbach’s *α* of 0.936. Standardized factor loadings ranged from 0.758 to 0.942, all above 0.50, confirming adequate item reliability. Model fit indices suggested a good fit (*χ*^2^ = 4.70, df = 2, CFI = 0.999, TLI = 0.996, RMSEA = 0.05, SRMR = 0.01).

#### 3.2.3 Innovation behavior

The Innovation Behavior variable was measured using the Innovation Behavior Scale developed by Scott and Bruce (1994), which includes 9 items assessing idea generation, promotion, and implementation. An example item is “I proactively propose new ideas and drive their implementation,” aligning with the scale’s focus on actionable innovation. The scale demonstrated high internal consistency, with Cronbach’s *α* values of 0.896, 0.902, and 0.920 for different factors. Standardized factor loadings ranged from 0.845 to 0.908, all above 0.50, supporting the reliability of the items. Model fit indices indicated an acceptable fit (*χ*^2^ = 48.82, df = 24, CFI = 0.99, TLI = 0.99, RMSEA = 0.05, SRMR = 0.02).

#### 3.2.4 Emotional intelligence

The Emotional Intelligence variable was assessed using the Wong and Law Emotional Intelligence Scale (WLEIS) (Wong and Law, 2002), which captures four dimensions: self-emotional appraisal, others’ emotional appraisal, regulation of emotions, and use of emotions. A representative item is “I usually understand the reasons behind my feelings,” reflecting the self-emotional appraisal dimension. Cronbach’s α for the four dimensions ranged from 0.944 to 0.951, indicating excellent internal consistency. Standardized factor loadings ranged from 0.843 to 0.943, all above 0.50. Model fit indices indicated reasonable fit (*χ*^2^ = 582.59, df = 84, CFI = 0.94, TLI = 0.93, RMSEA = 0.11, SRMR = 0.03).

#### 3.2.5 Control variables

To ensure the robustness of our analysis and accurately assess the factors influencing innovation behavior, we controlled for several key demographic variables based on established research. Sex has been shown to impact innovative tendencies, with previous studies suggesting potential differences in creativity and problem-solving styles between men and women (Baer and Kaufman, 2008). We treated sex as a categorical variable, coding 0 for female and 1 for male. Age has also been identified as a significant factor, as younger employees may bring fresh perspectives and adaptability, while older employees might leverage their experience to contribute to innovation (Ng and Feldman, 2013). Both work experience and work hours were included as continuous variables, as longer tenures and greater time investment in R&D roles have been positively correlated with innovation outcomes (Axtell et al., 2000; Janssen, 2000).

Education level plays a crucial role in shaping an individual’s capacity for innovation, with advanced degrees often associated with enhanced problem-solving skills and a deeper knowledge base (Cohen and Levinthal, 1990). We categorized education into bachelor’s, master’s, and doctoral levels to capture these variations. Job title was controlled as well, with positions ranging from junior to senior roles, as hierarchical differences in organizations often influence decision-making autonomy and access to resources for innovative tasks (Amabile et al., 2004). Additionally, unit size was included, as larger teams or organizational units may provide more resources and diversity of ideas, whereas smaller teams might foster closer collaboration (Van de Ven, 1986). We classified unit size into five categories, ranging from less than 50 to over 1,000 employees.

By including these control variables, we aimed to account for the potential demographic and organizational influences on innovation behavior, ensuring a more accurate analysis of the hypothesized relationships.

### 3.3 Ethical considerations

All participants were informed about the voluntary nature of their participation, and confidentiality was assured throughout the data collection process. Ethical approval was obtained from the relevant institutional review board (IRB) prior to the start of the study. Informed consent was obtained from all participants, and they were informed of their right to withdraw from the study at any time.

### 3.4 Data analysis

Before conducting the main analysis, we assessed the reliability and validity of the measurement scales using confirmatory factor analysis (CFA), a widely recognized tool for validating latent constructs (Hair et al., 2013). Key model fit indices such as chi-square (χ^2^), degrees of freedom (df), CFI, TLI, RMSEA, and SRMR were examined to evaluate the goodness-of-fit of the measurement model. These indices were used to ensure that the measurement model adequately represented the data, and that the standardized factor loadings met the recommended threshold of 0.50, which is essential for establishing construct validity.

Following the reliability and validity checks, we employed both preemptive and *post hoc* strategies to control for potential common method bias (CMV). In the preemptive phase, anonymity was guaranteed to reduce social desirability bias, and reverse-coded items and multiple Likert scales (7-point and 5-point) were used to diversify response patterns (Podsakoff et al., 2003). For post hoc detection, the marker variable technique was applied, along with additional CFA to check for CMV, ensuring that bias was not a significant issue in the data (Lindell and Whitney, 2001; Podsakoff et al., 2012).

After confirming the reliability of the measurement model and controlling for CMV, we proceeded with descriptive statistics and correlation analyses to explore the relationships among the main variables—openness, flow experience, innovation behavior, and emotional intelligence. Hierarchical regression analyses were then conducted to test the curvilinear mediation effect of flow experience and the moderating effect of emotional intelligence on the relationship between openness and innovation behavior (Hayes, 2018). Importantly, although interaction terms are often correlated with their constituent variables, Hayes (2017) emphasizes that this is a statistical inevitability, not a flaw, and that traditional concerns about multicollinearity in moderation analysis are largely misplaced. This approach allowed us to rigorously test the hypothesized relationships and interactions between the variables (Preacher and Hayes, 2008).

All data processing and statistical analyses were carried out using SPSS 26.0, Mplus v8.3, and PROCESS v4.0. SPSS was used for descriptive analyses, bivariate correlations, and regression modeling. PROCESS macro was applied for mediation and moderated mediation analysis with 5,000 bootstrap samples. Confirmatory factor analysis (CFA) was performed using Mplus to evaluate the measurement model and test for common method bias.

## 4 Results

### 4.1 Common method bias

In the *post hoc* phase, Harman’s single-factor test was conducted to assess the extent of CMV. The results indicated that the first principal component accounted for 43.29% of the variance, which is below the commonly accepted 50% threshold, suggesting that CMV was not a significant concern in the data (Podsakoff et al., 2003; Fuller et al., 2016). Furthermore, the marker variable technique was applied, and the correlations between the marker variable and other variables ranged from −0.03 to 0.03. These low and non-significant correlations further confirmed that CMV was unlikely to have influenced the results (Lindell and Whitney, 2001). The combination of preemptive strategies, such as anonymity assurance and varied questionnaire design, along with *post hoc* tests like Harman’s single-factor test and the marker variable technique, ensured that the data quality was not substantially compromised by common method bias. To effectively control for common method bias (CMV) in this study, both preemptive and *post hoc* strategies were employed. In the preemptive phase, anonymity assurance was used to minimize the impact of social desirability bias, ensuring that participants’ responses were more objective (Podsakoff et al., 2003). Additionally, the questionnaire design included reverse-coded items, as well as a combination of 7-point and 5-point Likert scales, to prevent uniform response patterns and further mitigate potential method bias (Fuller et al., 2016; Podsakoff et al., 2012). In the post hoc phase, Harman’s single-factor test and the marker variable technique were applied. The Harman’s single-factor test revealed that the first principal component accounted for 43.29% of the variance, which is below the 50% threshold, suggesting that CMV was not a major concern (Fuller et al., 2016; Podsakoff et al., 2003). Additionally, the marker variable technique revealed very low and non-significant correlations between the marker variable and the other variables (ranging from −0.03 to 0.03), indicating no significant presence of CMV (Lindell and Whitney, 2001). Overall, the combination of anonymity assurance and diverse questionnaire design in preemptive control, along with Harman’s single-factor test and the marker variable technique in post hoc detection, ensured that the data quality was not significantly compromised by CMV.

### 4.2 Descriptive statistics and correlations

Table 2 presents the descriptive statistics and correlations, showing significant positive relationships between innovation behavior and key variables like openness, emotional intelligence, and flow experience. All variables demonstrated acceptable normality (skewness: −0.92 to 1.15; kurtosis: −0.45 to 1.31).

**Table 2:** Descriptive statistics and correlations.

Variable	M	SD	1	2	3	4	5	6	7	8	9	10
1. Sex	1.25	0.43										
2. Age	3.08	0.89	0.12*									
3. Education level	3.61	0.61	0.10*	0.13**								
4. Work experience	2.76	1.55	0.11*	0.92**	0.08							
5. Title	2.43	1.32	−0.01	0.51**	0.05	0.52**						
6. Unit size	3.81	1.43	−0.07	−0.34**	−0.003	−0.27**	−0.02					
7. Work hours	2.88	0.68	0.05	0.23**	0.13**	0.18**	0.16**	−0.07				
8. Openness	4.29	1.20	−0.01	−0.11*	−0.13**	−0.16**	−0.12*	−0.02	−0.01			
9. Emotional Intelligence	3.89	0.71	−0.10*	−0.13**	−0.04	−0.15**	−0.17**	0.02	−0.06	0.49**		
10. Flow experience	3.97	0.74	−0.01	−0.04	−0.08	−0.12*	−0.07	−0.03	−0.06	0.65**	0.50**	
11. Innovation Behavior	3.86	0.45	−0.05	−0.02	−0.15**	−0.12*	−0.04	−0.02	0.03	0.51**	0.681**	0.60**

**p < 0.01, *p < 0.05.

### 4.3 Nonlinear mediation effect of flow experience

To test the nonlinear mediation effect of flow experience (FE) between openness (OP) and creative behavior (CB), we employed a stepwise regression approach combined with the bootstrap method for robust estimation of mediation effects. The stepwise regression involved two models: the first model regressed flow experience on openness and control variables, while the second model included flow experience, its squared term (FE^2^), and the control variables to predict creative behavior, testing for a curvilinear mediation effect. Additionally, we used bootstrap resampling (1,000 iterations) to estimate the confidence intervals of the direct, indirect, and total effects of openness on creative behavior through flow experience. Control variables included sex, age, education level, work experience, job title, unit size, and work hours.

#### 4.3.1 Stepwise regression results

We employed a stepwise regression method to examine the nonlinear mediation effect (Table 3). After controlling for sex, age, education, work experience, title, unit size, and work hours, Model 1 showed that openness (OP) had a significant positive effect on flow experience (FE) (*β* = 0.39, *p* < 0.001), indicating that higher openness is significantly associated with higher levels of flow experience, thus supporting the first half of the mediation path. Model 2 further tested the second half of the mediation path, revealing that flow experience had a significant positive effect on innovation behavior (*β* = 1.41, *p* < 0.001), while the squared term of flow experience (FE^2^) showed a significant negative effect (*β* = −0.16, *p* < 0.001), confirming a nonlinear relationship. This suggests that moderate levels of flow enhance innovation behavior, but excessive flow may reduce it, supporting the presence of an inverted U-shaped relationship. These findings demonstrate that openness influences flow experience, which in turn affects innovation behavior in a nonlinear manner. All models exceeded Cohen’s benchmark of *f*^2^ ≥ 0.35 for large effect sizes, as defined by Cohen (2013), with Model 4 showing an extraordinary effect size of *f*^2^ = 2.85.

**Table 3:** Regression analysis results of the moderated nonlinear mediation effect test.

Variable	Model 1		Model 2		Model 3		Model 4	
	Flow experience (FE)		Innovation behavior (IB)		Flow experience (FE)		Innovation behavior (IB)	
	B	SE	B	SE	B	SE	B	SE
(Intercept)	1.75***	0.27	0.164	0.267	1.89***	0.490	−3.13***	0.54
Openness (OP)	0.39***	0.02	0.103***	0.016	0.15	0.093	0.05***	0.01
Flow Experience (FE)			1.405***	0.120			2.50***	0.34
FE^2^			−0.159***	0.016			−0.26***	0.05
Emotional Intelligence(EI)					0.05	0.13	1.37***	0.15
OP x EI					0.04	0.02		
FE x EI							−0.47***	0.09
FE^2^ x EI							0.05***	0.01
sex	0.01	0.06	0.05	0.03	−0.043	0.06	0.002	0.03
age	0.24**	0.08	0.19***	0.05	0.261**	0.08	0.18***	0.034
education	−0.01	0.04	−0.07**	0.02	−0.029	0.04	−0.08***	0.02
work_experience	−0.14**	0.05	−0.10***	0.03	−0.144**	0.04	−0.11***	0.02
title	0.01	0.02	0.003	0.01	0.030	0.02	0.02*	0.01
unit_size	−0.001	0.02	0.004	0.01	0.002	0.02	−0.01	0.01
work_hours	−0.08*	0.04	0.02	0.02	−0.059	0.04	0.01	0.02
R-squared	0.43		0.52		0.48		0.74	
Adjusted R-squared	0.42		0.51		0.48		0.73	
Cohen’s *f^2^*	0.76		1.07		0.93		2.85	
*F*	44.460***		49.830***		43.380***		101.100***	

***p < 0.001, **p < 0.01, *p < 0.05.

#### 4.3.2 Bootstrap results for nonlinear mediation

**Figure 2:**
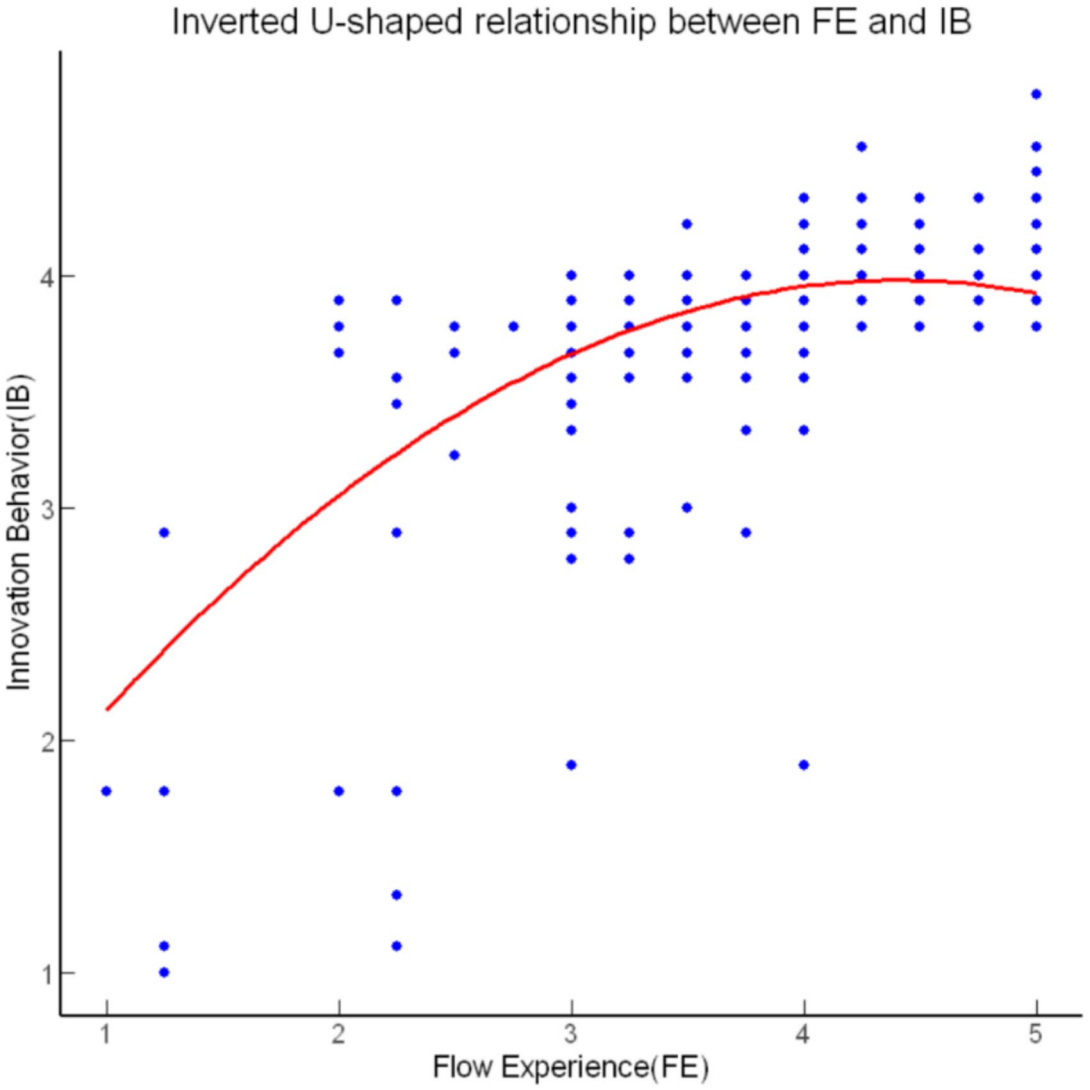
Inverted U-shaped relationship between FE and IB.

The bootstrap analysis provided further evidence for the nonlinear mediation effect of flow experience. As shown in Table 4, the indirect effect of openness on creative behavior through flow experience was significant (Estimate = 0.05, SE = 0.01, 95% CI [0.03, 0.08]), indicating that flow experience partially mediates this relationship. The direct effect of openness on creative behavior remained significant (Estimate = 0.10, SE = 0.02, 95% CI [0.06, 0.15]), confirming that openness directly influences creative behavior even after accounting for the mediation effect. The total effect of openness on creative behavior was also significant (Estimate = 0.15, SE = 0.02, 95% CI [0.12, 0.19]). These results support the existence of a nonlinear mediation effect, wherein openness influences creative behavior through flow experience in a curvilinear manner (Figure 2).

**Table 4:** Bootstrap results for nonlinear mediation effect.

Effect	Estimate	SE	CI_lower	CI_upper
Direct effect	0.10	0.02	0.06	0.15
Indirect effect	0.05	0.01	0.03	0.08
Total effect	0.15	0.02	0.12	0.19

### 4.4 Moderating effect of emotional intelligence

#### 4.4.1 No moderation on the first path (OP → FE)

After controlling for variables such as sex, age, and education, the analysis investigating the moderating effect of emotional intelligence (EI) on the relationship between openness (OP) and flow experience (FE) revealed no significant interaction effect (Model 3). Specifically, the interaction term between openness and emotional intelligence (OP x EI) was not significant (*B* = 0.04, *p* = 0.07), exceeding the conventional significance threshold of 0.05. Additionally, neither EI (*B* = 0.05, *p* = 0.72) nor the interaction term demonstrated significant direct effects on flow experience. These results indicate that emotional intelligence does not significantly moderate the relationship between openness and flow experience.

#### 4.4.2 Significant moderation on the second path (FE → CB)

In contrast, the results of Model 4 revealed that emotional intelligence (EI) significantly moderated the nonlinear relationship between flow experience (FE) and innovation behavior (IB) (Table 3). Specifically, the interaction between the squared term of flow experience and emotional intelligence (FE^2^ x EI) was significant (*B* = 0.05, *p* < 0.001), supporting the hypothesized moderating effect. Additionally, the interaction term between flow experience and emotional intelligence (FE x EI) was also significant (*B* = −0.47, *p* < 0.001), indicating that emotional intelligence plays a crucial role in shaping both the linear and nonlinear relationships between flow experience and innovation behavior. These findings suggest that emotional intelligence moderates the impact of flow experience on innovation behavior in both its linear and nonlinear forms, confirming the presence of a significant moderating effect.

**Figure 3:**
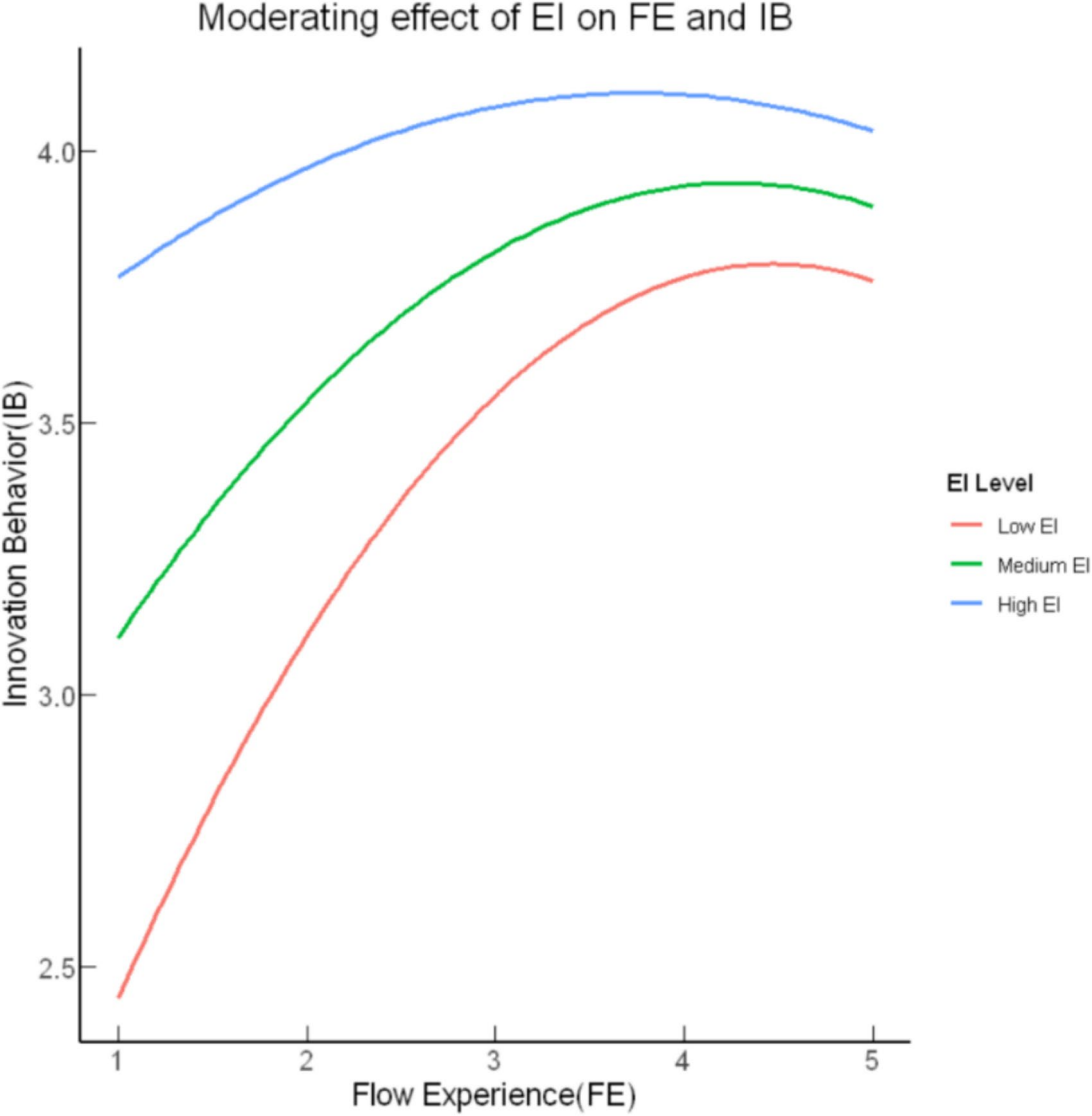
Inverted U-shaped relationship between FE and IB at different levels of EI.

By constructing a simple effects plot to examine the relationship between flow experience (FE) and innovation behavior (IB) at varying levels of emotional intelligence (EI) (Figure 3), we identified distinct patterns based on EI levels. Specifically, the results indicate that individuals with lower EI display a more pronounced inverted U-shaped relationship, where the positive effects of flow on innovation behavior peak earlier and decline sharply as flow increases. Conversely, those with higher EI demonstrate a more gradual rise in innovation behavior, with a slower and less severe decline at higher levels of flow. This suggests that higher EI facilitates better regulation of flow, helping to sustain its positive impact on innovation behavior even at elevated levels. These findings highlight the significant moderating role of EI in shaping the nonlinear relationship between flow experience and innovation behavior, providing a more comprehensive understanding of how EI influences creative performance in response to varying degrees of flow.

## 5 Discussion

This study aimed to examine the mediating and moderating roles of flow experience and emotional intelligence (EI) in the relationship between openness and innovation behavior among R&D professionals (Figure 1). The results partially support the proposed hypotheses, providing valuable insights into how flow experience and EI interact to influence creativity and innovation. Specifically, the study confirms the nonlinear mediating effect of flow experience and highlights the significant moderating role of EI in the flow-innovation relationship. These findings highlight the interplay between individual traits and psychological states. They also offer practical implications for enhancing innovation within R&D teams.

### 5.1 Hypothesis 1: nonlinear mediation role of flow experience

The results of this study confirm that flow experience mediates the relationship between openness and innovation behavior in a nonlinear manner. Specifically, our findings are consistent with Activation Theory, which posits that optimal activation enhances creativity, while excessive activation may hinder it (Montani and Vandenberghe, 2020). This inverted U-shaped relationship between flow experience and innovation behavior underscores the importance of balancing engagement levels during complex tasks. Moderate levels of flow significantly boosted innovation behavior, suggesting that when individuals achieve an optimal state of engagement, they are most productive in generating creative solutions (Ivancovsky et al., 2024).

The diminishing returns of excessive flow on innovation behavior can be explained by cognitive narrowing, where individuals become overly focused on specific tasks, thereby losing the broader perspective needed for innovative thinking. This phenomenon is well-documented in the literature, indicating that higher levels of flow can cause individuals to overlook critical environmental cues, limiting their ability to adapt and explore alternative solutions (McHugh, 2016; Rietzschel et al., 2016). The nonlinear mediation effect observed in this study reveals how varying levels of flow experience influence the relationship between openness and innovation behavior, illustrating the complex ways in which openness fosters innovation through flow.

These findings regarding the nonlinear mediation effect of flow experience offer valuable theoretical insights for fostering innovation-driven environments. Specifically, organizations can use these insights to design creative tasks that balance challenge and skill. Tasks that are too simplistic may fail to engage employees, while overly complex tasks can lead to frustration and cognitive overload, ultimately hindering innovation. Furthermore, setting clear and adaptive innovation goals is essential, as they provide direction while allowing flexibility, enabling employees to explore novel pathways to achieve creative outcomes. A supportive work environment is also crucial, as it ensures employees feel psychologically safe to experiment, reflect, and collaborate, thereby sustaining their engagement and creativity. Additionally, leadership practices must align with these principles, as managers play a critical role in identifying and maintaining optimal flow states among team members. Lastly, organizations should implement policies such as structured breaks and collaborative opportunities to help employees maintain balance and avoid burnout, ultimately ensuring sustained innovation.

### 5.2 Hypothesis 2a: emotional intelligence as a moderator between openness and flow experience

The results indicate that emotional intelligence (EI) did not significantly moderate the relationship between openness and flow experience. This finding challenges the theoretical expectation that individuals with higher EI are better equipped to channel their openness into deep engagement with complex tasks (Gagné et al., 2022; Ryan et al., 2019). One possible explanation for this is the multifaceted nature of flow initiation, which seems to be more influenced by extrinsic factors—such as the work environment, task structure, and immediate feedback—than by individual emotional capabilities. While openness provides the intrinsic motivation to explore new challenges, contextual factors may have a stronger influence on whether flow states are achieved, potentially reducing the moderating role of EI.

Additionally, the findings suggest that while EI plays a crucial role in managing emotional states and interpersonal interactions, its influence on the cognitive mechanisms underlying flow initiation may be indirect or context-dependent. This aligns with Self-Determination Theory (SDT), which highlights the importance of autonomy, competence, and relatedness in fostering engagement (Ryan et al., 2019). Research has shown that job characteristics, such as task clarity and meaningful feedback, are critical in facilitating flow experiences, sometimes outweighing the influence of personal traits like emotional intelligence (Ivancovsky et al., 2024). Future research could explore how EI interacts with these contextual factors to shape flow experiences, potentially revealing how EI indirectly supports engagement in novel and complex tasks.

### 5.3 Hypothesis 2b: emotional intelligence as a moderator in flow experience-innovation behavior relationship

The findings highlight a significant moderating effect of emotional intelligence (EI) on the nonlinear relationship between flow experience and innovation behavior. This underscores the pivotal role of EI in helping individuals sustain high levels of creativity even in situations where engagement fluctuates (Figure 3). Specifically, individuals with high EI can mitigate the potentially detrimental effects of excessive flow, such as cognitive narrowing, by maintaining cognitive flexibility and continuing to innovate productively even when absorbed in a task. This suggests that EI functions as a key psychological resource, balancing emotional regulation and cognitive engagement—an essential factor for sustaining innovation in complex professional settings.

The moderating role of EI in the flow-innovation relationship reflects its critical contribution to managing both cognitive and emotional processes during creative tasks. Drawing on the broaden-and-build theory of positive emotions, EI helps individuals maintain emotional regulation, which in turn broadens cognitive flexibility and prevents the cognitive narrowing that can result from excessive flow (Fredrickson, 2001). This adaptability enables high-EI individuals to continue innovative thinking by balancing focused attention with the exploration of alternative solutions (Rakei et al., 2022). Moreover, EI promotes integrative thinking, which is crucial for reconciling conflicting ideas and approaching challenges with balanced perspectives, thus overcoming the rigidity often associated with intense flow states (Goleman, 1996). Beyond individual benefits, EI also plays a crucial role in supporting collective innovation by enhancing interpersonal dynamics and shared emotional regulation. This ensures that team-based flow experiences are both resilient and productive, even in high-pressure environments (Darvishmotevali et al., 2018). These findings emphasize the multifaceted role of EI in navigating complex creative processes, both at the individual and team levels, positioning it as a cornerstone for sustained innovation.

This study provides valuable evidence to refine frameworks guiding R&D innovation, highlighting the transformative role of emotional intelligence (EI) in cultivating organizational and team innovation climates and advancing researcher development. To optimize task design in R&D, organizations should strike a balance between engagement and flexibility, taking into account emotional intelligence factors like emotional regulation and interpersonal skills. Tasks that integrate reflective opportunities and collaborative problem-solving can help sustain creativity under high cognitive demands while fostering psychological safety and promoting open communication—both of which are crucial for team-wide innovation. Additionally, the study advocates for integrating EI development into professional training programs for researchers. Tailored programs focusing on emotional management and teamwork skills can better prepare R&D personnel to handle high-stress challenges effectively. Furthermore, collaborative simulations and structured feedback mechanisms can help enhance adaptability, communication skills, and resilience, empowering researchers to thrive in dynamic, innovation-driven environments.

Although emotional intelligence (EI) did not significantly moderate the relationship between openness and flow in our study, field interviews with training managers from five R&D-intensive companies revealed that EI remains a key focus in innovation-oriented talent development. These organizations have integrated EI into various initiatives, such as conflict simulation workshops, emotional reflection tools in agile teams, and leadership coaching programs targeting interpersonal awareness.

One illustrative case comes from a robotics R&D company that implemented weekly “emotional status check-ins” within its engineering teams. While EI may not directly enhance flow for every individual, the team leaders used emotional awareness to adjust task difficulty and communication style in real time. This aligns with our finding that contextual factors may overshadow individual EI in influencing flow, especially in structured, high-pressure settings. Yet, the company reported a higher incidence of sustained engagement and creative output after introducing the practice—suggesting that EI can indirectly foster flow by shaping a psychologically responsive team environment.

### 5.4 Limitations and future directions

While this study offers meaningful insights into the interplay between personality, flow, and innovation-related outcomes, some limitations deserve consideration to guide future research. One limitation relates to the use of a cross-sectional design, which restricts the ability to make causal inferences. Although the proposed relationships were theoretically grounded and statistically supported, it remains unclear whether the observed effects unfold over time or in a particular sequence. A longitudinal or experimental design would provide stronger evidence of causality and could reveal how these dynamics evolve (Maxwell and Cole, 2007; Spector, 2019).

Another potential concern involves common method bias (CMV), given that all data were collected via self-reports at a single point in time. We took steps to minimize this risk—both through careful survey design and *post hoc* statistical checks like Harman’s single-factor test and the marker variable technique. While these procedures suggested that CMV was not a major issue, it’s still possible that some bias remains. Future studies might consider using multi-source or time-separated data to strengthen the robustness of findings (Fuller et al., 2016; Podsakoff et al., 2003).

A further area that invites deeper exploration is the non-significant moderating effect of emotional intelligence (EI) on the relationship between openness and flow. One explanation may lie in the role of contextual or environmental factors. In settings where autonomy is low or organizational constraints are high, even individuals with high openness and emotional intelligence may find it difficult to enter a flow state. This suggests that EI may not function uniformly across all environments, and that its impact could be contingent on contextual factors like task structure, job control, or psychological safety (Barrick et al., 2015). Investigating these conditions more explicitly would offer a richer understanding of when and how personality traits translate into optimal experience.

Additionally, the current research does not incorporate a cross-cultural perspective, which constitutes another important limitation. The relationships between personality, flow, and innovation may vary across different cultural contexts. Factors such as cultural values and social norms may influence the mechanisms by which openness and emotional intelligence affect flow. Future research could conduct cross-cultural replications to verify the generalizability of findings like nonlinear flow dynamics, clarify the moderating effects of culture, and enhance the universal applicability and theoretical breadth of the research conclusions.

Taken together, these limitations point to promising directions for future research. A more dynamic, multi-method, and context-sensitive approach—ideally incorporating longitudinal designs, environmental moderators, and cross-cultural explorations—would help clarify the nuances of how openness, emotional intelligence, and flow interact in real-world settings and refine the understanding of these relationships.

## 6 Conclusion

This study elucidates the dynamic interplay between openness to experience, flow experience, and emotional intelligence (EI) in shaping innovative behavior within R&D contexts. By integrating Flow Theory, Activation Theory, and EI into a unified framework, the research reveals two critical mechanisms: first, flow experience nonlinearly mediates the relationship between openness and innovation, exhibiting an inverted U-shaped pattern where moderate flow optimizes creativity, while excessive flow impedes it; second, EI moderates the curvilinear relationship between flow and innovation, buffering the detrimental effects of high flow intensity by enhancing cognitive flexibility and emotional regulation. These findings challenge the linear assumptions prevalent in prior literature, demonstrating that optimal innovation arises not merely from heightened engagement but from a balanced interplay of psychological states and individual competencies.

Theoretically, this work advances innovation research by introducing a novel integrative model that reconciles personality traits (openness), transient psychological states (flow), and emotional competencies (EI), offering a nuanced explanation of how and when openness translates into creative outcomes. Empirically, it is the first to validate the nonlinear mediation of flow and the moderating role of EI in this context, providing actionable insights for organizational practices. Practically, the study underscores the need for organizations to design tasks that maintain optimal flow through tailored challenge-skill balance, while investing in EI development programs to equip R&D personnel with tools to regulate intense flow states and sustain innovation under pressure. The case of the robotics R&D company further illustrates how contextual adjustments—such as real-time emotional check-ins—can amplify innovation outcomes by aligning task demands with individual capabilities. Collectively, these contributions highlight the value of harmonizing cognitive engagement with emotional resilience, offering a roadmap for cultivating sustainable innovation ecosystems in complex, high-stakes environments.

## Data Availability

The original contributions presented in the study are included in the article/supplementary material, further inquiries can be directed to the corresponding author.
